# Contributing Factors of Diabetes Mellitus among Patients with Gout (Results of the Long-Term Prospective Study)

**DOI:** 10.1134/S1607672923700321

**Published:** 2023-10-13

**Authors:** O. V. Zheliabina, M. S. Eliseev, S. I. Glukhova, E. L. Nasonov

**Affiliations:** 1 Nasonova Research Institute of Rheumatology, Moscow, Russia; 2 Sechenov First Moscow State Medical University, Ministry of Health Care of Russian Federation (Sechenov University), Moscow, Russia

**Keywords:** gout, type 2 diabetes mellitus, uric acid

## Abstract

It is assumed that the risk of developing type 2 diabetes mellitus (T2DM) in patients with gout is influenced by both generally accepted risk factors and factors related to gout. The aim of the study was to evaluate the impact of various risk factors for T2DM in patients with gout. A total of 444 patients (49 women, 395 men) ≥18 years old with gout and without DM were included. The duration of observation was 5.66 [2.69; 7.64] years. To identify the factors associated with the risk of developing T2DM, multivariate logistic regression was used, which included sex; T2DM in relatives; insufficient physical activity; unbalanced diet; age  ≥ 45 years; ≥4 attacks per year; presence of tophi; BMI ≥30 kg/m^2^; allopurinol, febuxostat, glucocorticoids, diuretics, metformin, colchicine; GFR < 60 mL/min/1.73 m^2^; serum uric acid level (sUA) ≥ 420 µmol/L and  ≥ 480 µmol/L. T2DM developed in 108 (24.3%) patients. According to the multivariate model, the presence of ≥4 attacks of arthritis per year increased the risk of T2DM (OR = 5.23; 95% CI: 2.98–9.19; *p* = 0.0001); presence of tophi (OR = 2.61; 95% CI: 1.50–4.54; *p* = 0.001); sUA ≥ 480 µmol/L (OR = 2.26; 95% CI: 1.02–5.00; *p* = 0.144); diuretics (OR = 2.35; 95% CI: 1.19–4.64; *p* = 0.014). Febuxostat (OR = 0.31; 95% CI: 0.11–0.84; *p* = 0.022) and metformin (OR = 0.49; 95% CI: 0.21–1.16; *p* = 0.107) reduced the risk of developing T2DM. Risk of T2DM in patients with gout is associated with high incidence of arthritis attacks, MK ≥ 480 μmol/L, hypertension, diuretic use, and febuxostat and metformin reduces risk.

Type 2 diabetes mellitus (T2DM) is one of the most urgent problems of the last few decades, the seventh most common cause of death in patients in the world [1]. The danger of the growing prevalence of T2DM lies, among other things, in serious complications associated with it [2].

The rapid increase in the incidence of T2DM is associated not only with the obesity epidemic but also with an increase in the proportion of the aging population, socioeconomic development, urbanization, high-calorie diets, and reduced physical activity. In general, the increased risk of T2DM is due to a combination of a number of genetic and metabolic factors. Non-modifiable factors include ethnicity, family history, previous gestational diabetes, and older age; the main modifiable factors include obesity, unbalanced diet, physical activity levels, and smoking [3].

The rapidly growing prevalence of gout is also undeniable: in just 10 years, a 60% increase in the prevalence of gout or hyperuricemia (HU) was identified [4]. Many risk factors that contribute to the onset or progression of gout were described, including age, male sex, obesity, alcohol, and various medications [5, 6].

Possibly, the almost twofold increase in the frequency of T2DM in patients with gout may be due to the presence of a large number of common risk factors for the development of these two diseases [7–9]. However, the influence of the factors inherent in gout itself (HU and chronic microcrystalline inflammation) also is not ruled out. For example, the association of HU and gout with insulin resistance syndrome (metabolic syndrome (MS)) was noted shortly after its description by Reaven in 1988 [10, 11] and continues to be discussed, in particular, from the standpoint of potential possibilities for correcting carbohydrate metabolism disorders using urate-lowering therapy (ULT) [12]. For example, recent studies showed that an increase in the level of uric acid (UA) in serum for every 1 mg/dL was accompanied by an increase in the risk of T2DM by 17% [13, 14].

However, prospective studies aimed at studying the risk factors for the development of T2DM, which could explain the causes for its high prevalence in patients with gout, have not yet been performed. The aim of our work was to assess the impact of various risk factors for type 2 diabetes mellitus in patients with gout on the basis of the results of long-term prospective follow-up.

The prospective single-center study included patients with gout who came to the Nasonova Research Institute of Rheumatology.

The inclusion criteria were age over 18 years and the diagnosis of gout that met the criteria by Wallace et al. [15]. The exclusion criteria were the presence of diabetes and pregnancy.

A total of 541 gout patients were screened. In 97 of them, T2DM was diagnosed at the first visit, and they were not included in the study. Thus, 444 patients with gout and without DM were included in the single-center prospective study. Data for each patient during the visits were entered into an individual registration card, dynamic monitoring was performed at least once every 2 years.

The 1999 World Health Organization criteria were used to confirm the diagnosis of T2DM [16].

During the visits, an anamnesis was taken and patients were examined. The following anthropometric parameters were assessed: height, body weight, waist circumference (WC), and body mass index (BMI). Obesity was diagnosed and its degree determined by BMI: BMI from 18.5 to 24.9 kg/m^2^ was regarded as norm; BMI from 25 to 29.9 kg/m^2^ corresponded to overweight; and BMI ≥ 30 kg/m^2^ corresponded to obesity [17]. Abdominal obesity (AO) was diagnosed at WC ≥94 cm in men and ≥80 cm in women. The presence and number of subcutaneous tophi, the number of gouty attacks over the past year, and the number of affected joints during the disease were determined.

Laboratory study included determination of serum glucose, creatinine, uric acid, C-reactive protein (CRP), and glycosylated hemoglobin.

The results were statistically processed on a personal computer using the parametric and nonparametric statistics methods in the Statistica 12.0 software (StatSoft Inc., United States). For qualitative traits, absolute and relative values (*n*, %) are presented; for quantitative traits, the median and the 25th and 75th percentiles. When two independent groups were compared for quantitative traits, the Mann–Whitney test was used; for comparison of qualitative traits, the χ^2^ test was used. The correlation between traits was assessed using Spearman’s rank correlation test (*r*). Differences were considered statistically significant at *p* < 0.05.

To identify the factors associated with the development of T2DM in patients with gout, multivariate logistic regression was used. The model includes the following factors: gender; the presence of T2DM in relatives; insufficient physical activity; unbalanced diet; age ≥45 years; ≥4 gout attacks per year; the presence of tophi; BMI ≥30 kg/m^2^; the presence of arterial hypertension (AH); taking allopurinol, febuxostat, glucocorticoids (GCs), diuretics, antihypertensive drugs, metformin, and colchicine; glomerular filtration rate (GFR) <60 mL/min/1.73 m^2^; and serum UA level ≥420 µmol/L and ≥480 µmol/L. Signs in which the odds ratio (OR) had *p* < 0.15 were considered as associated with the T2DM development.

During the follow-up period, T2DM developed in 108 out of 444 (24.3%) patients included in the study. During the follow-up period, 34 patients died and five patients were lost to follow-up.

The clinical characteristics of the patients with gout included in the study, as well as the comparative characteristics of patients who had and did not have T2DM by the end of the follow-up period, are presented in Table 1.

**Table 1.** Characteristics of gout patients included in the study

Parameters	All patients (*n* = 444)	T2DM developed (*n* = 108)	T2DM did not develop ( *n* = 297)	R*
Men, *n* (%)	395 (88.9)	96 (88.8)	265 (89.2)	0.92
Age, years, М ± σ	51.2 ± 11.7	52.8 ± 10.9	49.7 ± 11.9	0.02
Duration of observation (years), Me[25th; 75th percentile]	5.7 [2.7; 7.6]	5.3 [2.2; 7.9]	6 [2.8; 7.7]	0.38
Duration of gout (years) Me[25th; 75th percentile]	5.6 [1.6; 7.2]	5.1 [1.4; 7]	5.6 [1.9; 7.2]	0.55
Presence of tophi, *n* (%)	176 (39.6)	64 (59.3)	89 (29.9)	0.001
≥4 arthritis attacks per year, *n* (%)	181 (40.8)	73 (67.6)	94 (31.6)	0.001
Number of arthritis attacks per year Me[25th; 75th percentile]	5 [2, 7]	5 [2, 9]	4 [2, 6]	0.004
The number of affected joints during the disease ≥5, *n* (%)	339 (76.4)	86 (79.6)	220 (74.1)	0.25
Compliance with a hypopurine diet, *n* (%)	88 (19.8)	27 (25)	53 (17.8)	0.11
Smoking, *n* (%)	101 (22.7)	26 (24.1)	65 (21.9)	0.64
Alcohol consumption ≥20 units per week, *n* (%)	174 (42.9)	47 (43.5)	127 (42.7)	0.89
BMI, kg/m^2^, М ± σ	29.97 ± 4.8	30.11 ± 5.03	30.03 ± 4.77	0.89
BMI ≥25 kg/m^2^, *n* (%)	380 (85.6)	95 (87.9)	252 (84.8)	0.43
BMI ≥30 kg/m^2^, *n* (%)	213 (47.9)	54 (50)	146 (49.1)	0.88
Abdominal obesity (Waist ≥80 cm in women, ≥94 cm in men), *n* (%)	253 (56.9)	96 (88.9)	264 (88.9)	0.99
DM in 1st-line relatives, *n* (%)	94 (21.2)	43 (39.8)	55 (18.5)	0.001
DM in 1st and 2nd line relatives, *n* (%)	99 (22.3)	53 (49.1)	76 (25.6)	0.0001
Insufficient physical activity, *n* (%)	327 (73.6)	80 (74.1)	196 (65.9)	0.12
Unbalanced diet, *n* (%)	344 (77.5)	82 (75.9)	202 (68.1)	0.12
Laboratory indices Accompanying illnesses				
CRP, mg/L, Me [25th; 75th percentile]	14.7 [4.9; 19.7]	13.33 [4.2;18.8]	14.9 [4.9;17.5]	0.74
CRP ≥5 mg/L, *n* (%)	318 (71.6)	74 (68.5)	213 (71.7)	0.53
Glucose (mmol/L), М ± σ	5.42 ± 0.82	5.33 ± 0.8	5.5 ± 0.8	0.2
Cholesterol (mmol/L), М ± σ	5.65 ± 1.37	5.5 ± 1.3	5.7 ± 1.4	0.22
Cholesterol ≥5 mmol/L, *n* (%)	302 (68.0)	70 (64.8)	205 (69)	0.42
Triglycerides ≥2.5 mmol/L, *n* (%)	173 (38.9)	48 (44.4)	111 (37.4)	0.2
Creatinine (µmol/L), М ± σ	101.53 ± 31.15	100.9 ± 28.15	101.6 ± 32.7	0.85
GFR <60 mL/min/1.73 m^2^, *n* (%)	48 (10.8)	12 (11.1)	32 (10.7)	0.92
UA (µmol/L), М ± σ	500.0 ± 111.7	541.23 ± 115.1	480.05 ± 102.6	0.0001
UA<300 µmol/L, *n* (%)	90 (22.2)	13 (12.54)	77 (26.6)	0.003
UA ≥480 µmol/L, *n* (%)	228 (56.3)	77 (71.3)	151 (50.8)	0.0002
UA ≥420 µmol/L, *n* (%)	348 (78)	82 (76)	236 (79)	0.14
UA ≥600 µmol/L, *n* (%)	72 (17.8)	37 (34.3)	35 (11.8)	0.0002
Accompanying illnesses				
Ischemic heart disease, *n* (%)	124 (27.9)	37 (34.3)	69 (23.2)	0.003
Arterial hypertension, *n* (%)	361 (81.3)	94 (87)	233 (78.4)	0.05
Heart failure, *n* (%)	51 (11.5)	12 (11)	27(9)	0.54
Myocardial infarction, *n* (%)	36 (8.1)	12 (11)	17 (5.7)	0.006
Stroke, *n* (%)	19 (4.3)	6 (5.5)	9(3)	0.23
Urolithiasis, *n* (%)	210 (47.3)	61 (56.5)	149 (50.2)	0.26
Medical therapy				
Urate-lowering drugs, *n* (%)	326 (73.4)	80 (74.1)	231 (77.8)	0.74
Allopurinol, *n* (%)	280 (63.1)	73 (67.6)	190 (63.9)	0.5
Febuxostat, *n* (%)	46 (10.4)	7 (6.5)	41 (13.8)	0.042
Metformin, *n* (%)	59 (13.3)	10 (9.5)	49 (16.5)	0.06
Canakinumab, *n* (%)	19 (4.3)	5 (4.6)	13 (4.4)	0.9
Diuretics, *n* (%)	85 (19.1)	30 (27.7)	44 (14.8)	0.003
Glucocorticoids, *n* (%)	164 (36.9)	51 (47.2)	108 (36.4)	0.047
Antihypertensive drugs, *n* (%)	361 (81.3)	79 (73.1)	150 (50.5)	0.0001

T2DM, type 2 diabetes mellitus; BMI, body mass index; CRP, C-reactive protein; GFR, glomerular filtration rate; UA, uric acid; IHD, ischemic heart disease; AH, arterial hypertension; CHF, chronic heart failure; MI , myocardial infarction; ACCI, acute cerebrovascular accident; ICD, urolithiasis.* Differences between patients who developed and did not develop type 2 diabetes mellitus.

As can be seen from the presented data, the patients who developed T2DM during the follow-up period were older, and their relatives had a higher incidence of T2DM than in the families of patients without T2DM. In addition, the patients with T2DM were more likely to have AH and subcutaneous tophi; they had ≥4 attacks of arthritis per year, serum UA levels ≥480 µmol/L and ≥600 µmol/L, and more often took diuretics and GCs. At the same time, the level of UA <300 µmol/L was detected more often in patients without T2DM.

To identify the risk factors associated with the development of T2DM in patients with gout, we used a multivariate logistic regression model. The sensitivity of the model was 46.3%, specificity 92.3%, and multiple determination coefficient *R*^2^ = 0.373. The factors associated with the risk of T2DM are presented in Table 2.

**Table 2.** Factors associated with an increase or decrease in the risk of T2DM

Factors	OR	95%CI	*p*
Male	1.01	0.42–2.47	0.977
DM relatives	1.69	0.61–4.68	0.311
Insufficient physical activity	0.85	0.44–1.67	0.644
unbalanced diet	1.48	0.78–2.82	0.231
Age ≥ 45 years	1.25	0.65–2.40	0.505
≥4 arthritis attacks per year	5.23	2.98–9.19	0.0001*
The presence of tophi	2.61	1.50–4.54	0.001*
BMI ≥ 30kg/m^2^	1.33	0.77–2.32	0.307
Arterial hypertension	1.82	0.83–4.02	0.137*
GFR < 60 mL/min/1.73 m^2^	0.22	0.03–1.87	0.165
UA ≥ 420 µmol/L	1.61	0.79–3.25	0.182
UA ≥ 480 µmol/L	2.26	1.02–5.01	0.044*
Medical therapy			
Colchicine	0.76	0.44–1.31	0.33
Diuretics	2.35	1.19–4.6	0.014*
Metformin	0.49	0.21–1.16	0.107*
Allopurinol	1.26	0.69–2.29	0.456
Febuxostat	0.31	0.11–0.84	0.022*
Glucocorticoids	1.12	0.64–1.99	0.69

OR, odds ratio; 95% CI, 95% confidence interval; DM, diabetes mellitus; BMI, body mass index; GFR, glomerular filtration rate; UA, uric acid. * Signs for which OR had *p* < 0.15 were considered as associated with the development of type 2 diabetes.

The number of attacks of arthritis ≥4 per year was associated with a 5-fold increase in the risk of development of T2DM; in the presence of tophi, it increased 2.6 times; at a level of MK ≥ 480 µmol/L, 2.3 times; during antihypertensive therapy, 2.6 times; when taking diuretics, 2.4 times. Conversely, taking febuxostat and metformin was accompanied by a 3.2- and 2.02‑fold decrease in the risk of T2DM development.

The association between HU, gout, and MS components, including carbohydrate metabolism disorders, was shown in many studies, including population studies [18–20]. Evidence is accumulating that gout increases the risk of developing MS and directly T2DM. For example, Choi et al. [21] in a prospective study examined the association between gout and the risk of developing T2DM over 6 years in 11351 men at high cardiovascular risk and found that men with gout were at a higher risk of developing T2DM regardless of other known factors. Kim et al. [22] showed that gout was associated with an increased risk of T2DM compared with osteoarthritis after adjusting for potential distorting factors. However, previous studies analyzed the impact of T2DM risk factors in cohorts of patients with GU and gout, but not the factors specifically related to gout, such as disease severity, which we did for the first time.

The cause for the influence of gout on the risk of T2DM is not fully understood. Possible explanations include systemic inflammation, which can worsen insulin sensitivity, as well as intracellular urate synthesis-related inhibition of the enzyme AMP kinase, which may play the key role in the genesis of both HU and hyperglycemia in the case of high fructose consumption [23]. Other studies show that increased xanthine oxidase activity may be associated with the development of hyperinsulinemia, insulin resistance, and, as a result, T2DM, regardless of the serum level of urate [24, 25]. Rho et al. [7], on the basis of the results of their population study, proposed several potential pathophysiological mechanisms that may be responsible for the frequent coexistence of gout and T2DM, including chronic inflammation inherent in gout, the effects of sex hormones, taking certain medications, and genetic factors. Lai et al. [26] found that gout and T2DM have a number of common genetic factors that increase the probability of combination of these two diseases.

In addition, the presence of common risk factors in gout and T2DM (including obesity, hypertension, dietary habits, lipid metabolism disorders, and taking diuretics), may also be important. For example, we have previously shown that, in patients with gout who had T2DM, the conventional risk factors for T2DM such as AH and taking diuretics are found more often [27]. In addition, we noted that gout combined with T2DM proceeded more severely: in such patients, subcutaneous tophi were detected more often, and the serum UA level was higher. Previously, in a small study, it was shown that gout that was accompanied by non-severe T2DM was associated with higher serum UA levels before the start of ULT. Arthritis attacks in such patients develop more often, and the number of affected joints is greater than in the patients with gout without T2DM [28].

These data suggested that, if the observed association between gout and T2DM is causal, then the incidence of T2DM should be directly correlated with the severity of gout, in particular, with the manifestations of the disease such as the frequency of arthritis attacks, the presence of tophi, and uncontrolled HU. Conversely, effective treatment of gout with adequate ULT may be a possible way to reduce this risk.

Our results support this hypothesis. The results of logistic regression analysis showed that, in addition to hypertension and taking diuretics, HU, a high frequency of arthritis attacks, and the presence of subcutaneous tophi were associated with the development of T2DM, whereas heredity burdened by T2DM, insufficient physical activity, and obesity had no statistically significant effect on the risk of T2DM development in this cohort of patients. For example, a serum UA level ≥480 µmol/L increased the likelihood of T2DM development 2.3 times, and in patients who strictly controlled the level of uricemia (maintaining a serum UA concentration <300 µmol/L), T2DM developed 2.1 times less frequently. Moreover, the presence of subcutaneous tophi was associated with a 2.6-fold increase in the risk of T2DM, and a high frequency of arthritis attacks (≥4 attacks per year) was associated with its 5-fold increase.

Our data on the effect of the uricemia level are consistent with the results of other studies [14, 29, 30]. For example, a Finnish study of 557 overweight or obese individuals with impaired glucose tolerance showed that baseline UA was a predictor of T2DM after adjusting for age, sex, blood pressure, BMI, serum triglyceride and creatinine levels, physical activity, and diet (*p* = 0.037) [30].

Another equally important result is our data on the effect of drug therapy on the risk of T2DM development.

In particular, we showed that taking febuxostat was associated with a 3.2-fold reduction in the risk of T2DM, whereas no such association was found for allopurinol. This can be explained by the fact that febuxostat should be considered as a more effective urate-lowering drug compared to allopurinol, and the likelihood of achieving the target serum UA level in the case of its prescription is higher [31–33]. The beneficial effect of ULT on the manifestations of MS may be associated with a decrease in the severity of chronic inflammation and insulin resistance. For example, Meng et al. [34] reported that the treatment with febuxostat reduced UA levels and insulin resistance in gout patients.

Although HU is a risk factor for both T2DM development and increased mortality in patients with T2DM and there is an opinion that lowering the level of UA may be one of the potential goals of therapy in patients with T2DM [35, 36], data of few studies in which the antidiabetogenic effect of urate-lowering drugs was investigated are controversial. For example, 41 patients with T2DM were randomized for treatment with allopurinol at a dose of 100 mg/day or placebo for 14 days [37]. However, this study showed no significant reduction in fasting blood glucose or in glycosylated hemoglobin levels in either group. Moreover, Chang et al. [38], after analyzing data on taking allopurinol and benzbromarone in 29765 gout patients included in the Taiwan national database and twice as many controls found that taking both drugs was associated with an increased risk of developing T2DM. This association was more pronounced in the patients who took drugs for a long time and at a higher cumulative dose, whereas low doses, conversely, were associated with a reduced risk in individuals older than 50 years [38]. These authors point out a number of limitations of the study, in particular, low adherence to ULT, lack of data on the level of UA, other laboratory data, physical activity, and diet, which could affect the results of the study. As in our study, there was no increase in the risk of T2DM in gout patients with increasing age; moreover, it was found that patients younger than 50 years had a greater risk of T2DM than those over 50 years of age.

On the other hand, several randomized trials showed that lowering UA levels with allopurinol can compensate for insulin resistance in asymptomatic HU patients [39, 40].

Another drug that should be mentioned in the context of the results of this study is metformin, which is associated with a 2.02-fold reduction in the risk of T2DM. The preventive effect of metformin on the T2DM development in patients with elevated fasting glucose was reported previously [41]. However, it was not known whether this effect would be realized in gout patients. It can be assumed that the reduction in the risk of T2DM development was associated not only with the direct hypoglycemic effect of metformin due to an increase in the absorption and metabolism of glucose in tissues and the suppression of gluconeogenesis in the liver, but also with the pleiotropic effects of the drug, which can be realized in gout. For example, according to Vazirpanah et al. [42], metformin reduced the production of proinflammatory cytokines (primarily interleukin-1β) by immune cells induced by sodium monourate crystals in vitro by inhibiting mTOR (mammalian target of rapamycin), whose expression by monocytes, as well as B and T cells increased in the presence of monourate crystals. The next phase of the study in patients with gout showed that taking metformin in combination with allopurinol decreased almost twice the incidence of arthritis attacks compared with allopurinol alone. In another small study in patients with gout and insulin resistance, taking metformin at a dose of 1500 mg/day was accompanied by a statistically significant decrease in serum UA level, fasting insulin concentration, and HOMA insulin resistance index [43]. Currently, a number of analyses discuss the possibility of wider use of metformin in gout patients [44, 45]; however, studies that could confirm the feasibility of such treatment are still insufficient.

Thus, the obtained results confirm the association of factors directly related to gout (in particular, serum UA level, the frequency of arthritis attacks, and the presence of tophi) with the risk of T2DM, which may be the cause for the frequent combination of these diseases. Effective treatment of gout with the use of adequate ULT can be considered a potential method for preventing carbohydrate metabolism disorders in such patients. The results obtained in this study indicate the need for further research on this issue.
